# STIM1 Controls the Focal Adhesion Dynamics and Cell Migration by Regulating SOCE in Osteosarcoma

**DOI:** 10.3390/ijms23010162

**Published:** 2021-12-23

**Authors:** Yu-Shan Lin, Yi-Hsin Lin, MyHang Nguyen Thi, Shih-Chuan Hsiao, Wen-Tai Chiu

**Affiliations:** 1 Institute of Basic Medical Sciences, National Cheng Kung University, Tainan 701, Taiwan; kanaour@gmail.com; 2 Department of Biomedical Engineering, National Cheng Kung University, Tainan 701, Taiwan; wsp90020@gmail.com (Y.-H.L.); myhang.dhyd@gmail.com (M.N.T.); 3 Department of Hematology & Oncology, Saint Martin de Porres Hospital, Chiayi 600, Taiwan; annette.hsiao@msa.hinet.net; 4 Medical Device Innovation Center, National Cheng Kung University, Tainan 701, Taiwan

**Keywords:** STIM1, SOCE, focal adhesion, cell migration

## Abstract

The dysregulation of store-operated Ca^2+^ entry (SOCE) promotes cancer progression by changing Ca^2+^ levels in the cytosol or endoplasmic reticulum. Stromal interaction molecule 1 (STIM1), a component of SOCE, is upregulated in several types of cancer and responsible for cancer cell migration, invasion, and metastasis. To explore the impact of STIM1-mediated SOCE on the turnover of focal adhesion (FA) and cell migration, we overexpressed the wild-type and constitutively active or dominant negative variants of STIM1 in an osteosarcoma cell line. In this study, we hypothesized that STIM1-mediated Ca^2+^ elevation may increase cell migration. We found that constitutively active STIM1 dramatically increased the Ca^2+^ influx, calpain activity, and turnover of FA proteins, such as the focal adhesion kinase (FAK), paxillin, and vinculin, which impede the cell migration ability. In contrast, dominant negative STIM1 decreased the turnover of FA proteins as its wild-type variant compared to the cells without STIM1 overexpression while promoting cell migration. These unexpected results suggest that cancer cells need an appropriate amount of Ca^2+^ to control the assembly and disassembly of focal adhesions by regulating calpain activity. On the other hand, overloaded Ca^2+^ results in excessive calpain activity, which is not beneficial for cancer metastasis.

## 1. Introduction

Calcium (Ca^2+^) signaling is involved in various cellular events ranging from cell life to cell death, including cell proliferation, differentiation, migration, apoptosis, and necrosis [1]. A key mechanism that regulates Ca^2+^ concentration in non-excitable cells is store-operated calcium entry (SOCE), which delineates the depletion of Ca^2+^ in the endoplasmic reticulum (ER), leading to the influx of Ca^2+^ via the Ca^2+^ release-activated Ca^2+^ (CRAC) or transient receptor potential cation (TRPC) channels located on the plasma membrane [2], thus initiating the downstream Ca^2+^-related signaling pathways [3]. Stromal interaction molecules, including STIM1 and STIM2 (STIMs), which are ER Ca^2+^ sensors, forms clusters on the ER membrane when there is ER Ca^2+^ depletion and regulate Ca^2+^ homeostasis by activating CRAC or TRPC channels, such as Orai1 or TRPC1 [4,5]. STIM1 initiates SOCE response by sensing severe ER Ca^2+^ store depletion, while STIM2 detects mild-to-moderate store depletion, and thus it is thought of playing a housekeeping role to maintain ER Ca^2+^ concentration [6,7]. Recently, many studies have found that STIM1 is essential for cancer cell migration, invasion, and metastasis [8]. Some studies have proposed that STIM1-mediated SOCE facilitates cancer cell migration. STIM1 overexpression is associated with enhanced cell migration and invasiveness [9,10], while its knockdown decreases the cell migration and increases focal adhesion (FA) size and intensity, which could be considered an impediment to cell migration [11,12]. Contrastingly, some studies have identified STIM1 as a tumor suppressor because its overexpression in some specific types of cancers leads to cancer cell death or has no effects on the cells [13,14]. These findings suggest that STIM1 may have multiple regulatory mechanisms in different cancer cells [15,16], further implying the importance of Ca^2+^ homeostasis controlled by STIM1-mediated SOCE in cancer.

Cell migration is an essential physiological process that controls embryonic development, immune response, tissue homeostasis, and tumor progression [17]. It begins with morphological polarization, during which the leading edges and rear edges of the cells can be distinguished, followed by lamellipodia and filopodia protrusions at the leading edges [18]. During cell migration, the nascent small focal adhesive complexes at the leading edge gradually aggregate to increase in size and complexity and generate force to pull the cell body forward, causing the formation of mature FA proteins to stabilize the attachment [19], while the release of FA proteins at the rear edge results in the net movement of the cells. As a consequence, FA turnover is a crucial process in cell migration because directional migration requires the continuous assembly of FA proteins at the leading edge and disassembly at the rear edge. Ca^2+^ signaling is also involved in directional sensing, cytoskeleton redistribution, force generation, and FA dynamics in cell migration [20]. In particular, Ca^2+^ mediated by SOCE promotes FA turnover through Ca^2+^-dependent proteins, such as calpain. SOCE is known to regulate cell migration via FA dynamics.

Osteosarcoma is the most common primary malignant bone tumor in 10–24 year old children and young adults. Most patients with osteosarcoma easily die from cancer metastasis due to poor diagnosis. The five-year survival rate of these patients after lung metastasis is only 20–30%; therefore, the prevention of metastasis is the best strategy to be considered [21]. Many factors in the bone microenvironment are involved in metastasis, including hypoxia, acidosis, the existence of mesenchymal stem cells, the release of chemokines or anti-inflammatory cytokines by tumor-associated fibroblasts, and the activation of Ca^2+^-related signaling pathways [22]. Understanding the mechanisms underlying cell migration may help in exploring new strategies for the prevention or treatment of cancer metastasis. As mentioned above, STIM1 has been reported to regulate the cancer cell growth, proliferation, and migration, and it has been found to be upregulated in a variety of cancers [23,24]. In osteosarcoma cell lines, the overexpression of STIM1 promotes the cell viability and cell migration [25], and it also contributes to the chemoresistance [26]. Hence, targeting STIM1 to manipulate Ca^2+^ homeostasis may be a promising strategy to prevent cancer metastasis.

In this study, we investigated the effects of STIM1-mediated SOCE on cell migration by overexpressing different STIM1 variants in an osteosarcoma cell line. For the STIM1 variants used in this study, D76 (negatively charged aspartic acids) in the STIM1 EF-hand was mutated to a non-charged amino acid, alanine (A), resulting in a constitutively active form of the STIM1 variant. In addition, the deletion of the cytoplasmic ezrin–radixin–moesin (ERM) region, which can interact with Orai1 and TRPC1, makes it difficult for STIM1 to form puncta at the juxta membrane and bind with Orai1 or TRPC1, thus leading to a dominant-negative form (D76A-ΔERM) of the STIM1 variant [27,28]. By overexpressing these STIM1 variants, which display different activities in the U2OS cell line, our results adumbrate that STIM1-meditated SOCE affects FA dynamics by regulating the activity of calpain, thus having an impact on single-cell or collective cell migration.

## 2. Results

### 2.1. Changes of SOCE Levels in STIM1 Variants-Overexpressing U2OS Cells

**Figure 1:**
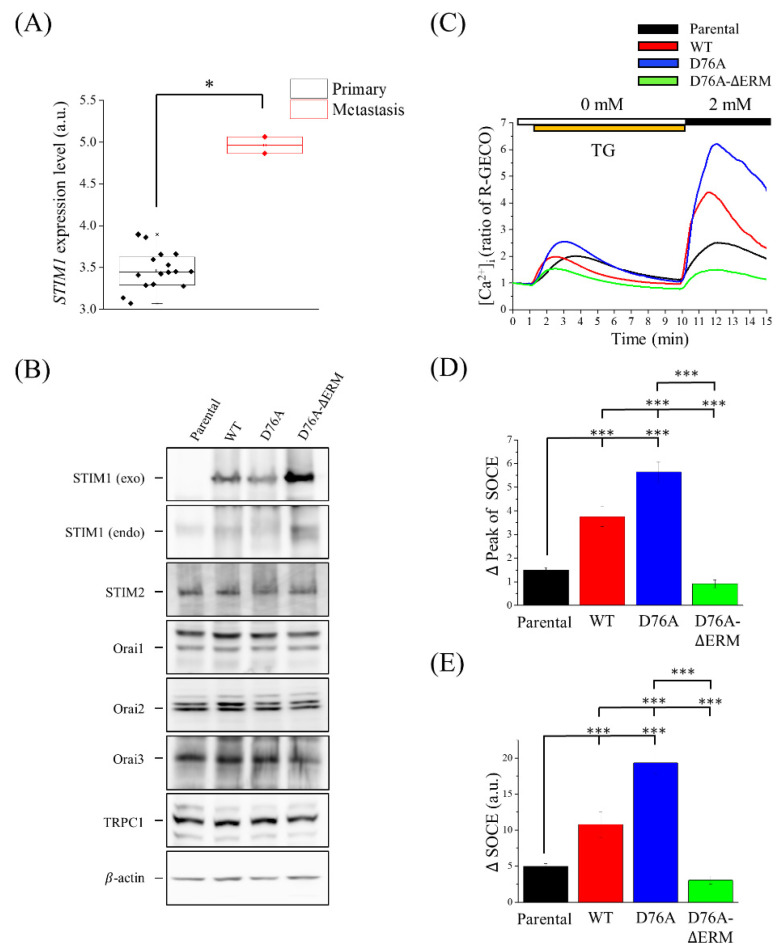
STIM1 variants regulate SOCE level in the U2OS osteosarcoma cell line. (**A**) The box plot represents *STIM1* gene expression levels in the primary or metastatic osteosarcoma cell lines. Data was analyzed from GEO dataset. (**B**) Cells were transfected with or without STIM1 variants. Antibodies against SOCE-related proteins, including exogenous and endogenous STIM1, STIM2, Orai1, Orai2, Orai3, and TRPC1, were used in the immunoblotting analysis. β-actin served as the internal control. (**C**) Representative result of changes in intracellular Ca^2+^ levels in the cells transiently transfected with R-GECO, of which fluorescence ratio intensity represented the Ca^2+^ level. Extracellular 2mM Ca^2+^ buffer was added at the tenth minute. Each trace was obtained by averaging data from 15–44 cells. TG, thapsigargin. (**D**) Quantification of the maximum peak of SOCE. Bars represent the mean ± standrad error of the mean (SEM). (**E**) Quantification of the area under SOCE curve which indicates SOCE-mediated total Ca^2+^ influx. Bars represent the mean ± SEM. * *p* < 0.05, *** *p* < 0.001 by Mann-Whitney test in (**A**), one-way analysis of variance (ANOVA) in (**D**) and (**E**). a.u., arbitrary unit.

We first analyzed the Gene Expression Omnibus (GEO) dataset to determine the relationship between STIM1 expression levels and cancer progression [29]. Cancer cells with metastatic ability showed higher STIM1 expression (Figure 1A). Consequently, we overexpressed different fluorescent protein-tagged STIM1 variants, including SIMT1-wild-type (WT)-enhanced green fluorescent protein (EGFP), STIM1-D76A-EGFP (constitutively active), and STIM1-D76A-ΔERM-enhanced yellow fluorescent protein (EYFP) (dominant negative) in U2OS osteosarcoma cell lines to further investigate whether cancer cell migration can be affected by manipulating SOCE levels through STIM1 activity. Immunoblotting showed that exogenous STIM1 had a higher expression than endogenous STIM1, and D76A-ΔERM variants had been found to upregulate the endogenous STIM1 compared to the other groups. The expression levels of different STIM1 variants in cells did not affect the expression levels of other SOCE-related component proteins (Figure 1B). To study the impact of different STIM1 variants on SOCE, we monitored the intracellular Ca^2+^ levels by transiently transfecting STIM1 variants into the USOS cell lines that stably expressed R-GECO, which is a genetic Ca^2+^ indicator with a red fluorescence emission [30], and analyzed the intracellular Ca^2+^ levels using a single-cell fluorometer. The intensity of R-GECO was quantified and represented as cytosolic Ca^2+^ concentration; therefore, when Ca^2+^ bound to R-GECO, the intensity of red fluorescence emission would increase. First, cells were incubated in a Ca^2+^-free buffer and treated with thapsigargin (TG) for 1 min to deplete the Ca^2+^ in the ER and induce the release of Ca^2+^ from ER, which in turn activates STIM1. The above process indicated the baseline physiological Ca^2+^ concentration and ER-stored Ca^2+^ in the cells. In the second step, extra Ca^2+^ was introduced into the extracellular solution, leading to SOCE Ca^2+^ influx (Figure 1C). Based on these results, WT and D76A variants increased the SOCE levels, while D76A-ΔERM variants decreased them compared to the cells that did not overexpress any STIM1 variants, the parental U2OS cells (Figure 1C–E). The quantification was performed by subtracting the highest R-GECO intensity from the lowest one, which suggested the largest Ca^2+^ influx at one specific moment (Figure 1D), or by integrating the area under the SOCE curve, which indicated the total Ca^2+^ influx (Figure 1E).

### 2.2. Localization of Different STIM1 Variants on the Cell Membrane

**Figure 2:**
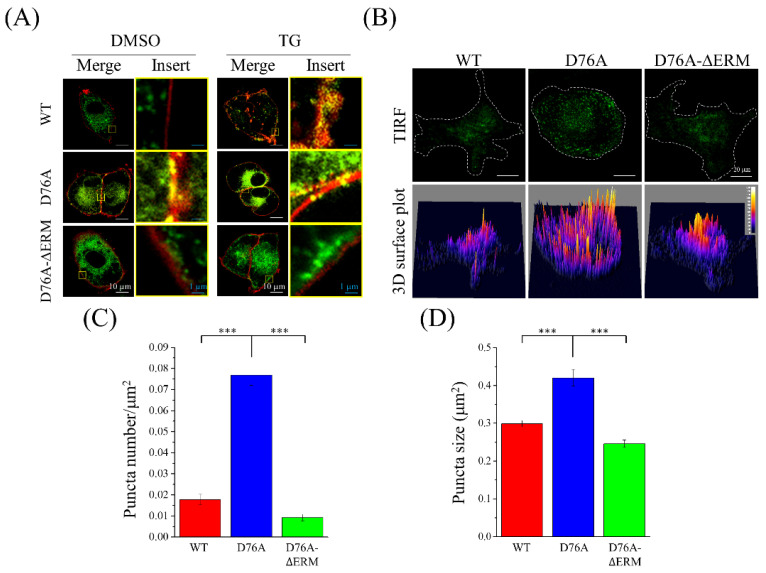
Localization of STIM1 variants on the plasma membrane. (**A**) The plasma membrane marker, ChR2-mCherry, was transiently transfected into the cells. Then, the cells were treated with dimethyl sulfoxide (DMSO) or 2 μM TG to trigger the SOCE response. Representative confocal images were shown with fixed exposure time. Green, STIM1; red, ChR2. (**B**) Upper panel: representative TIRF images were taken without Ca^2+^ depletion in the endoplasmic reticulum (ER). White dotted line represents the boundary of the cells. Lower panel: 3D surface plot of the fluorescent protein-tagged STIM1 variants in resting-state cells. (**C**,**D**) Quantification of number and size of STIM1 puncta from (**B**). *n* > 21 in every group. *** *p* < 0.001 by one-way ANOVA.

To observe the location of STIM1 in the cells, the membrane marker, mCherry-tagged channel rhodopsin 2 (ChR2), was transiently transfected in STIM1-overexpressing cells. WT variants co-localized with membrane markers only when SOCE was activated by TG; however, most of the D76A variants co-localized with ChR2, regardless of Ca^2+^ depletion from the ER. In contrast, D76A-ΔERM variants did not come into contact with the cell membrane even after TG treatment (Figure 2A). To further examine STIM1 activity, a total internal reflection fluorescence (TIRF) microscopy was used to avoid the interference of background fluorescence, and the activated STIM1 puncta at the juxta membrane could also be observed clearly (Figure 2B). Without ER Ca^2+^ depletion by TG, D76A variants formed the greatest number and size of puncta at the cell membrane compared to WT and D76A-ΔERM variants (Figure 2C,D), suggesting that D76A variants were always in an activated state and ready for SOCE response at any time.

### 2.3. Cells Expressing Different STIM1 Variants Show Distinct Cell Migration Abilities

**Figure 3:**
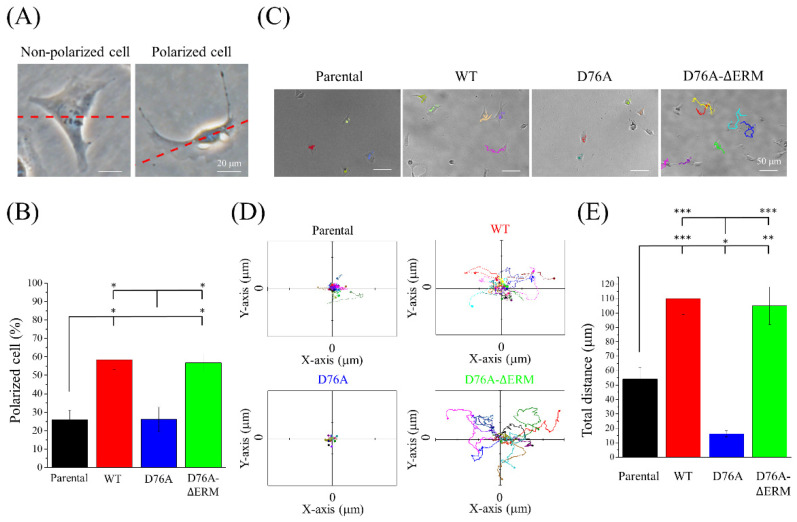
Impact of STIM1 variants on cell migration. (**A**) Phase contrast images showed the morphology of non-polarized and polarized cells. The extended lamellipodium at on side was displayed in polarized cells. (**B**) Quantification of polarized cell percentage under different STIM1 variant-expressed cells. Bars represent the mean ± SEM. (**C**) Migration trace of cells expressing different STIM1 variants was observed for 4 h by the mini-imaging system. The pictures were taken every 20 s. Representative images taken at 4 h were shown. Each cell was marked with a different color and at least 25 cells were recorded. (**D**) Trajectory images were obtained from the time-lapse images recorded in (**C**). Start position was set to 0. Each color represented the trace of a single cell. (**E**) Quantification of total distance from the time-lapse recording images. Bars represent the mean ± SEM. * *p* < 0.05, ** *p* < 0.01, *** *p* < 0.001 by one-way ANOVA.

**Figure 4:**
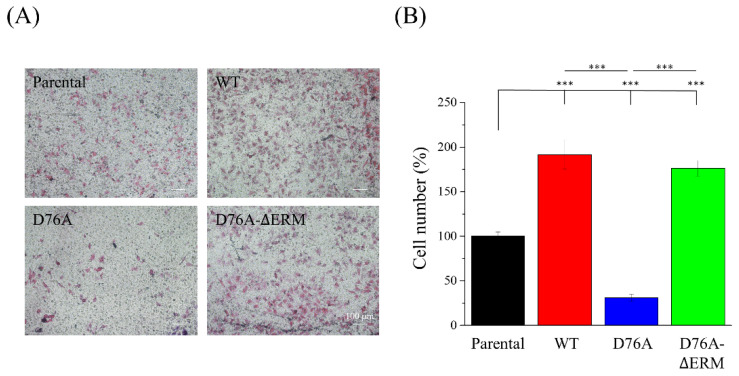
Constitutively active STIM1 decreases the single-cell migration ability. (**A**) Representative images of the transwell single-cell migration assay. Cells that migrated through 0.8 μm polycarbonate membrane were fixed and stained with the hematoxylin and eosin dye. (**B**) The percentage of migrated cells was quantified from three independent experiments. Bars represent the mean ± SEM. *** *p* < 0.001 by one-way ANOVA.

STIM1 mediates cell migration in various cancers. Therefore, we attempted to clarify whether the cancer migration ability is influenced by the STIM1-mediating SOCE response. A polarized cell was recognized by the extended lamellipodium on one side of the cell (Figure 3A). According to previous studies proposing that cancer cells with STIM1 upregulation display better cell migration ability, we hypothesized that cancer cells overexpressing constitutively active STIM1 (D76A form) would exhibit better migration abilities, while those with dominant negative STIM1 (D76A-ΔERM form) would show decreased migration abilities. Nevertheless, we surprisingly found that the percentage of polarized cell population was higher in the cells that overexpressed WT and D76A-ΔERM variants (Figure 3B), indicating that their potential migration ability was better than the others. In the time-lapse recording imaging, increased cell migration was observed in WT and D76A-ΔERM variants (Figure 3C), as displayed in the trajectory analysis obtained from the time-lapse imaging (Figure 3D). The quantification of the total distance also showed a significant elevation in WT and D76A-ΔERM variants; on the other hand, D76A variants hindered the 2D cell migration ability compared to the other STIM1 variants (Figure 3E). In the following experiment, we performed a transwell assay to test the 3D single-cell migration ability. Consistent with the time-lapse recording imaging, the number of cells that crossed over 8 μm pores increased if the cells expressed WT and D76A-ΔERM variants, and cells in which STIM1 is constitutively active exhibited defective single-cell migration ability (Figure 4A,B). In addition to single-cell migration ability, we also examined the collective cell migration using a wound healing assay (Figure S1A). Although WT and D76A-ΔERM variants did not increase the speed of wound closure compared to their parental variants, D76A variants dramatically impeded the collective cell migration ability (Figure S1B). The above results imply that an excessive SOCE response predominated by constitutively active STIM1 may retard the cell migration or invasion during cancer metastasis.

### 2.4. Focal Adhesion Dynamics Were Changed by STIM1 Overexpression

**Figure 5:**
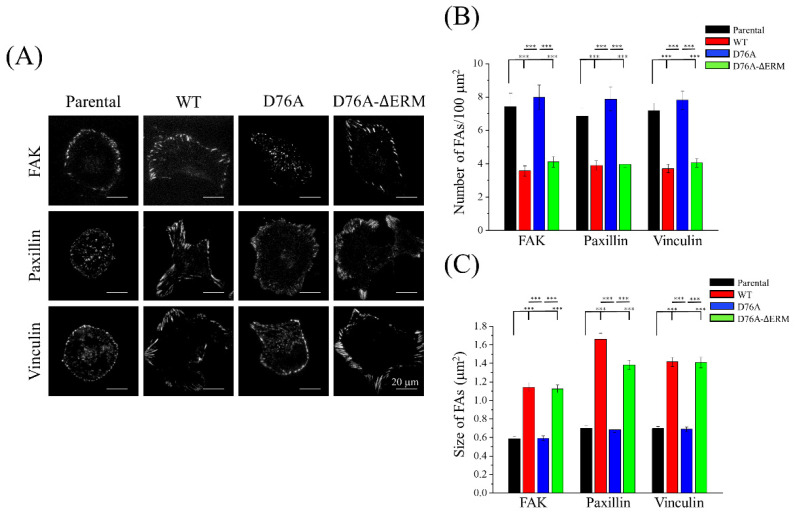
Overexpression of different STIM1 variants affects the focal adhesion dynamics. (**A**) Images of immunofluorescence-stained focal adhesion proteins, including FAK, paxillin and vinculin, were taken and observed under TIRF microscope. The exposure time was fixed in all of the images. (**B**,**C**) Quantification of the number and size of focal adhesion proteins in cells expressing different STIM1 variants. Bars represent the mean ± SEM. *** *p* < 0.001 by one-way ANOVA.

The assembly and disassembly of FA proteins are dynamically regulated during cell migration [31]. Based on the above results, we propose that the FA dynamics of D76A variants may be different from those of the other variants. The immunofluorescence staining images of FA proteins, such as FAK, paxillin, and vinculin, were observed under a TIRF microscope. In general, longer and larger FA proteins were located at the peripheral sites in cells expressing WT and D76A-ΔERM variants (Figure 5A). Similar to our migration assays, the number of FA proteins in D76A variants was higher than in other variants, while the size of FA proteins in D76A variants was smaller than in WT and D76A-ΔERM variants (Figure 5B,C), suggesting that cancer cells need appropriate FA dynamics to optimize their migration abilities. A faster assembly and disassembly of FA in D76A variants did not favor superior cell migration ability.

### 2.5. STIM1 Activation Meditated the Calpain Activity

**Figure 6:**
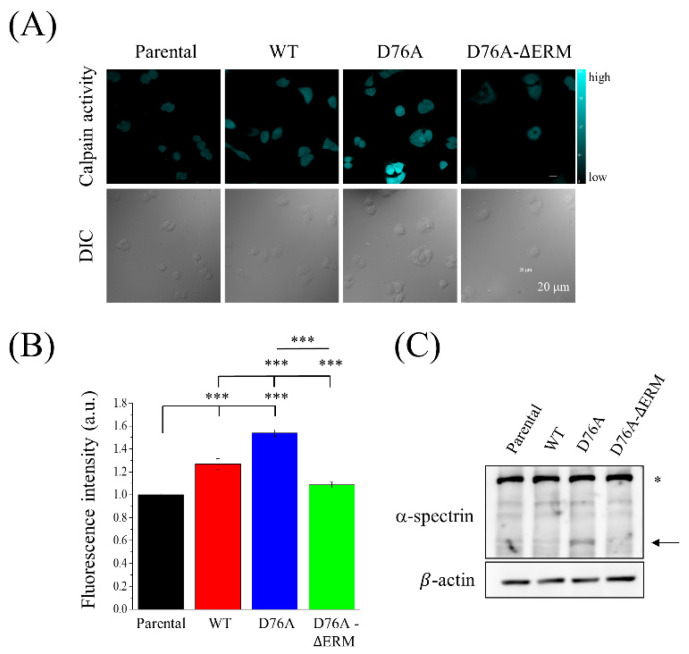
Calpain activity is influenced by the overexpression of different STIM1 variants. (**A**) Cells overexpressing different STIM1 variants were treated with t-BOC-LM-CMAC, which is an artificial fluorogenic calpain substrate, for 30 min. Confocal images showed the fluorescence intensity (upper panel) and the differential interference contrast (lower panel) of the cells. (**B**) Quantification of fluorescent intensity, which stood for calpain activity, from three independent experiments. Bars represent the mean ± SEM. *** *p* < 0.001 by one-way ANOVA. (**C**) Immunoblotting analysis using antibodies against the calpain substrate, α-spectrin. The stars and arrows indicate the intact and fragmented α-spectrin, respectively. β-actin served as the internal control.

Since calpain, a Ca^2+^-dependent protease, has been reported to promote FA degradation and affect cell migration [32,33], we hypothesized that an overloaded Ca^2+^ concentration caused by D76A variants may activate calpain to cleave FA proteins, thereby resulting in smaller FA proteins and a poor migration ability of cells. Therefore, we performed a calpain activity assay and found that the D76A variants significantly increased the calpain activity compared to the parental, WT, and D76A-ΔERM variants (Figure 6A,B). The immunoblotting analysis also showed that the expression levels of cleaved α-spectrin, which is the substrate of calpain, were elevated in D76A variants (Figure 6C). In conclusion, cells that overexpressed D76A variants displayed excessive SOCE levels, thus leading to calpain activation. Therefore, the cleavage of FA proteins by activated calpain makes it difficult for cancer cells to migrate to distant sites.

## 3. Discussion

In this study, we demonstrated how different SOCE levels induced by three different STIM1 variants affect FA dynamics and cell migration. After stably overexpressing STIM1 variants in the osteosarcoma U2OS cell line, we first examined how these variants influenced the SOCE function. As expected, the cells expressing D76A variants, which is the constitutively active form of STIM1, showed the highest SOCE levels, and they would bind to orai1 receptors even though there was no store depletion by TG treatment. Therefore, we hypothesized that D76A variants may increase the cell migration ability. In contrast to this assumption, we found that the cells exhibited defective cell migration upon the overexpression of D76A variants. The detailed mechanism is described as follows. The higher intracellular Ca^2+^ influx induced by D76A variants activates Ca^2+^-dependent protease, calpain, which cleaves the FA proteins. As a consequence, the number of FA proteins increases, and the size of FA proteins decreases, thus inhibiting the cell migration. These results were consistent with those of previous research. Some researchers have proposed that calpain can be activated mainly by the elevation of Ca^2+^ concentration [34], while others have reported that calpain modulates the adhesion dynamics by accelerating the proteolysis of FAK and vinculin [35,36]; however, the cleavage of paxillin by calpain increases cell adhesion and negatively regulates cell migration [37]. Moreover, it has been reported that the size of FA proteins can be used to predict the speed of cell migration, and larger, more elongated FA proteins have been shown in fast-moving cells [38].

High intracellular Ca^2+^ concentrations have been reported to be related to necrotic, apoptotic, and autophagic cell death. To prevent Ca^2+^ overload-induced cell death, there might be a compensatory mechanism to balance the Ca^2+^ levels in cells [39]. In our study, we found that cells overexpressing D76A variants easily died in a high-density culture (data not shown), indicating that the overexpression of the D76A mutant is lethal to cells, while the cells lived well in a relatively low to moderate density culture. Our Western blotting analysis showed that exogenous STIM1 expression levels were lower in constitutively active D76A variants than other variants, and D76A variants displayed the lowest expression levels of endogenous STIM1 (Figure 1B).

In our results, although the D76A-ΔERM variants presented the minimum level of SOCE, it showed a similar impact on cell migration as the WT variants. The alterations in focal adhesion dynamics and calpain activity between WT and D76A-ΔERM variants were also similar. The phenomena mentioned above may be due to the upregulation of endogenous STIM1 after the overexpression of D76A-ΔERM variants in cells (Figure 1B), and different time scales in intracellular Ca^2+^ measurement and migration assays may also be a concern. The measurement of SOCE was completed within 15 min, while the 2D single-cell migration, transwell, and wound healing assays lasted for 7, 16, and 24 h, respectively. Other possible reasons include the local Ca^2+^ pulses from TRP channels, or the rear-high and front-low intracellular Ca^2+^ gradient regulated by the Ca^2+^ ATPase of the plasma membrane [40]. Our previous study also showed that the knockdown of STIM1 upregulated STIM2 to compensate for the deficiency of SOCE in MEF (mouse embryonic fibroblast) cells [41]. Although there were no changes in STIM2 expression levels in U2OS cells expressing STIM1 or other STIM1 mutants (Figure 1B), the activity of STIM2 might be higher in D76A-ΔERM variants than in other variants.

One important thing that should be elucidated is that the similar phenomenon seen in WT and D76A-ΔERM variants does not mean that the effect of STIM1 on migration is independent of SOCE activation. As shown in Figure 1, we tested SOCE levels in different STIM1 variants-expressing cells by using TG, which depletes the ER Ca^2+^ stores and forces the activation of SOCE. However, this artificial stimulation is used to demonstrate the STIM1 function and the maximum capacity of SOCE, and thus it cannot be referred to reality. Without outside stimulation, STIM1 cannot be activated at any time under the overexpression of WT because there is no Ca^2+^ depletion in ER lumen, while SOCE is always activated in the D76A variant, leading to the increase of calpain activity and the acceleration of focal adhesion turnover, thus making the inhibition of cell migration. The D76A-ΔERM variant lacks the domain combined with SOC channels, so it does not exert the function of STIM1 to trigger SOCE, even if the STIM1 is in a state of constitutive activation. In a nutshell, it suggests that STIM1-meidated SOCE is involved in migration because the excessive influx of Ca^2+^ impedes the migration of D76A-expressing cells. On the other hand, even if the underlying mechanism of increasing migration ability in WT or D76A-ΔERM variants may not be the same, the results still imply that cancer cells possess the innate ability to regulate Ca^2+^ influx to increase their survival.

In response to mechanical and chemical signals, cells may change their morphology into front-to-rear polarity to facilitate directional migration [42]. Our results showed that there was an increasing population of polarized cells under the overexpression of WT and D76A-ΔERM variants. However, in the wound healing assay, the parental cells that did not overexpress any STIM1 variants showed similar results compared to cells expressing WT and D76A-ΔERM variants (Figure S1). This may be due to cell-cell contact, which allows the cells to affect the behavior of their neighbors and alter their front-to-rear polarity [43]. Collective cell migration requires the organization of cellular forces, cadherin-based or integrin-based adhesion complex, and the regulation of Rho family GTPases and actin dynamics [44], suggesting that the superior migration ability of parental cells observed in the wound-healing assay but not the transwell assay (Figure S1) might be related to the cell–cell adhesive interactions.

In conclusion, the overexpression of STIM1 with normal function in an osteosarcoma cell line increased the cell migration ability. Similarly, the presence of the dominant negative form of exogenous STIM1 also promoted cell migration, implying that cancer cells have the innate ability to regulate Ca^2+^ influx to benefit their survival. However, if the cells cannot control Ca^2+^ homeostasis under the overexpression of the constitutively active form of exogenous STIM1, the migration ability would be obstructed by the overloaded Ca^2+^ influx. In light of our in vitro findings, Ca^2+^ homeostasis may be an important factor that should be considered for the development of novel drugs targeting STIM1 for cancer treatment, and further in vivo study need to be performed in the future.

## 4. Materials and Methods

### 4.1. Cell Line and Cell Culture

Human bone osteosarcoma cell line (U2OS) cells provided by Dr. Yu-Chao Chang (School of Dentistry, Chung Shan Medical University, Taiwan) were cultured in low-glucose Dulbecco’s modified Eagle’s medium (DMEM, Simply Biologics, #CC130-1000, Taipei, Taiwan) supplemented with 10% fetal bovine serum (FBS; Gibco) and 1% penicillin-streptomycin (Simply Biologics, #CC502-0100). Cells were incubated in 5% carbon dioxide (CO_2_) at 37 °C. STIM1-WT-EGFP, STIM1-D76A-EYFP, and STIM1-D76A-∆ERM-EYFP constructs were gifts provided by Dr. Shmuel Muallem (National Institutes of Health, Bethesda, MD, USA) and were transiently transfected into the USOS cells for subsequent experiments.

### 4.2. Bioinformatics Analysis

The GEO2R web tool was used to analyze the GSE33458 dataset. All calculations were performed in the default settings. The results are presented as a table of genes that were ordered in the sequence of significance. *STIM1* gene IDs were found in the GEO datasheets. Entering the ID on the website revealed the profile graph and sample values of the *STIM1* gene expression level. The gene expression level was then plotted using the Origin9 software.

### 4.3. Single-Cell Ca^2+^ Measurement

U2OS cells stably overexpressing the cytoplasm-localized red Ca^2+^ indicator, R-GECO (U2OS-R-GECO), were used to measure the cytosolic Ca^2+^ levels. STIM1 variants were transiently transfected into the U2OS-R-GECO cell line. The emissive fluorescence intensity of R-GECO can be represented as the intracellular Ca^2+^ level. After seeding in 3-cm dishes, STIM1 variant-overexpressing U2OS-R-GECO cells were bathed in 2 μM TG (Sigma, #T9033, Saint Louis, MO, USA) 1 min after incubation with the Ca^2+^-free buffer. After 10 min, 2 mM Ca^2+^ buffer was used for TG treatment. Time-lapse images were recorded using a single-cell fluometer (TILL Photonics, Grafelfing, Germany). The total recorded period was measured for 15 min at 3 s intervals.

### 4.4. Western Blotting

Cell lysates were collected using an ice-cold modified radioimmune precipitation assay (RIPA; 150 mM sodium chloride (NaCl), 10 mM ethylene glycol tetraacetic acid (EGTA), 50 mM Tris-HCl (pH 7.5), 10% glycerol, 1% nonidet P-40 (NP-40), 0.5% sodium deoxyoholate, 0.1% sodium dodecyl sulfate (SDS), 0.004% sodium azide, Complete^TM^ protease inhibitor cocktail (Roche), 1 mM sodium orthovanadate (Na_3_VO_4_), 1 mM sodium fluoride, and 1 mM phenylmethanesulfonyl fluoride (PMSF)). The protein lysates were quantified using the Lowry protein assay (Bio-Rad). Proteins were separated through 5–15% SDS-polyacrylamide gel electrophoresis (PAGE). Electrophoretic blotting was performed to transfer the proteins onto the nitrocellulose paper. The membrane was blocked with 5% milk diluted in Tris-buffered saline (TBS) (0.4 M Tris, 1 M NaCl; pH = 7.6) and incubated with primary antibodies overnight at 4 °C. Immune complexes were detected using horseradish peroxidase-conjugated IgG antibodies (Jackson ImmunoResearch Laboratories, West Grove, PA, USA). Finally, ECL chemiluminescence was visualized using the ImageQuant LAS 4000 system (GE Healthcare Life Sciences, Pittsburgh, PA, USA). Primary antibodies against STIM1 (Abcam, #ab108994, Waltham, MA, USA), STIM2 (Cell signaling, #4917s, Beverly, MA, USA), Orai1 (Santa Cruz, #sc-377281, Santa Cruz, CA, USA), Orai2 (Santa Cruz, #sc- 376749), Orai3 (ProSci, #4215, Poway, CA, USA), TRPC1 (Abcam, #ab63074), α-spectrin (Abcam, #ab11751), and β-actin (Sigma, #A2066) were used in this study.

### 4.5. Confocal Microscopy

After seeding cells in 24-well plates and incubating overnight, we used Lipofectamine 3000 reagent and P3000 reagent (Invitrogen, Camarillo, CA, USA) to transfect the plasma membrane marker, ChR2-mCherry DNA plasmids, for 30 h. After successful transfection, we cultured the cells in 3 cm glass-bottom dishes and incubated them for 12 h for subsequent experiments. The cells were fixed with 4% paraformaldehyde (PFA; Alfa Aesar, #43368, Haverhill, MA, USA) for 20 min. Images were taken using a confocal microscope (FV-3000; Olympus, Tokyo, Japan), and 488 and 594 nm lasers were used to excite the fluorophores. Enlarged inserts were gated using FV10-ASW (Olympus, Tokyo, Japan) software to determine the co-localization of STIM1 and ChR2.

### 4.6. TIRF Microscopy and Image Analysis

To observe STIM1 puncta, the cells were fixed with 4% PFA (#43368; Alfa Aesar) for 20 min after seeding on 3 cm glass-bottom dishes. Images were taken with an Olympus IX81-ZDC inverted microscope with TIRF and differential interference contrast (DIC) microscopy. The number and size of STIM1 puncta were quantified using the ImageJ software. For focal adhesion profiles, cells were seeded on 3-cm glass-bottom dishes and fixed with 4% PFA (#43368, Alfa Aesar) for 20 min. The cells were then treated with 0.1% Triton-X for 10 min at room temperature. Primary antibodies such as anti-paxillin (BD Biosciences, #610051, Franklin Lakes, NJ, USA), anti-FAK (BD Biosciences, #610088), and anti-vinculin (Invitrogen, #700062) were added after the cells were incubated in CAS blocking solution (Invitrogen, #008120) for 1 h. The FA structures could be observed clearly in the 90–100 nm evanescent wave of the 561 nm laser. At least 30 cells were counted. The quantification of the number and size of FA proteins was performed using the ImageJ software.

### 4.7. Polarity Assay

To separate aggregate cells into single cells and induce cell polarization, the cells were suspended in fresh medium without FBS and penicillin-streptomycin. Then, 3.3 × 10^4^ cells were prepared in 1 mL medium with 1% BSA, followed by incubation in 5% CO_2_ for 1.5 h at 37 °C. The cells were then seeded on a fibronectin-coated 3 cm dish. After the cells were incubated for 3 h, images were captured using bright-field microscopy. The data were collected from three independent experiments.

### 4.8. Wound Healing Assay

Culture inserts (ibidi, GmbH) with two wells separated by a 500 μm-thick silicon wall were exposed to UV light before being placed into a 3 cm culture dish. Equal numbers of cells were seeded (2 × 10^5^ cells/100 μL) in two wells and incubated overnight. The inserts were removed after the cells were attached. Changes in gap were recorded at 0, 12, 18, and 24 h in phase-contrast mode with an Olympus inverted microscope. The data were collected from three independent experiments, and the wound closure area was analyzed using the ImageJ software.

### 4.9. 2D-Cell Migration Assay

The 3 cm dishes were coated with 10 μg/mL fibronectin for 2 h at 37 °C. At the same time, the cells were trypsinized and suspended in low-glucose DMEM containing 1% BSA for 1.5 h. After removing fibronectin from the dishes, we seeded the cells in coated dishes and incubated them for 3 h. The mini-image system (Lumascope 520; Etaluma) was placed in the incubator. Images were taken every 20 s for 4 h under a white light. The data were collected from at least 30 cells. The auto-tracking plugin in ImageJ was used to analyze the total distance of individual cell migration.

### 4.10. Transwell Assay

Transwell inserts and plates (6.5 mm insert, 24 well plate, 8 μm pore polycarbonate membrane; #3422; COSTAR) were rinsed with low-glucose DMEM for 30 min. The cells were suspended in the medium and seeded at a density of 2 × 10^4^ in the inserts. The cells were then treated with 10% FBS DMEM and incubated for 16 h. Then, the cells were fixed with 4% PFA for 2 min at room temperature. After air drying, the cells were stained with hematoxylin and eosin stain (Leica) for 15 min and washed with phosphate-buffered saline (PBS) three times. The cells were air-dried before taking pictures. The cell numbers were counted using an Olympus inverted microscope. The quantification and statistical analyses were performed using the Origin9 software.

### 4.11. Calpain Activity

The CMAC peptidase substrate, *t*-BOC-Leu-Met (7-Amino-4-Chloromethylcoumarin, *t*-BOC-L-Leucyl-L-Methionine amide), can be used to measure the calpain activity in live cells. The cleavage of t-BOC-Leu-Met produces blue fluorescence with an excitation/emission maximum of ~351/430 nm. To compare the calpain activity under the overexpression of STIM1 variants, the cells were incubated on a 3 cm glass-bottom dish and treated with 2 μM TG for 10 min followed by incubation with 10 μM t-BOC-LM-CMAC for 20 min. After the cells were washed twice with PBS, the buffer was immediately replaced with a 2 mM Ca^2+^ buffer. Ten pictures were captured using a confocal microscope (FV3000; Olympus). The data were collected from three independent experiments and quantified using the ImageJ software.

### 4.12. Statistical Analysis

All results are presented as the mean ± standard error of the mean (SEM). For a statistical analysis, a one-way analysis of variance (ANOVA) with Dunnett’s post-hoc test was performed using the Origin9 software. The Mann-Whitney test was used to analyze the GEO dataset. The statistical significance was set at *p* < 0.05.

## Data Availability

No applicable.

## Supplementary Materials

Supplementary materials can be found at https://www.mdpi.com/article/10.3390/ijms23010162/s1.
