# Supplementary material for: STIM1 Controls the Focal Adhesion Dynamics and Cell Migration by Regulating SOCE in Osteosarcoma
Source: Int J Mol Sci. 2021 Dec 23;23(1):162. doi: 10.3390/ijms23010162 (PMC8745645; doi:10.3390/ijms23010162)
Supplement: Supplementary file 1 [file ijms-23-00162-s001.zip › ijms-1480799-supplementary.pdf]

Supplementary information

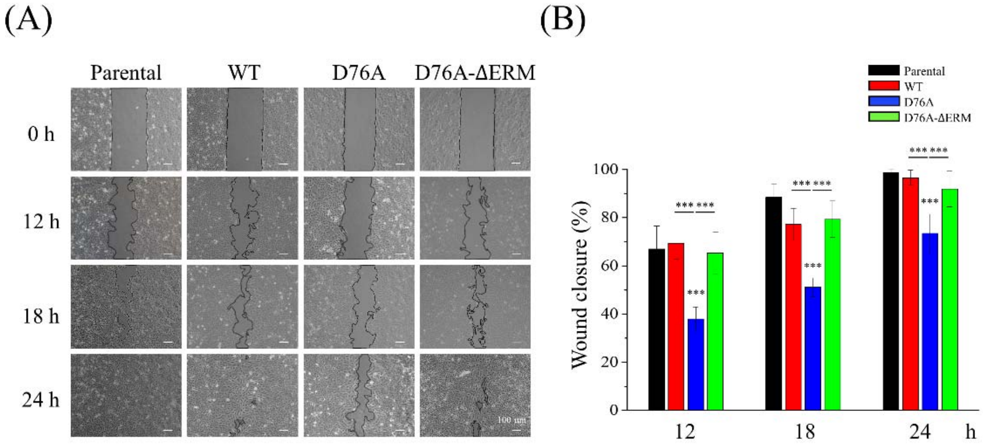

Figure S1. Different STIM1 variants result in different migration abilities in osteosarcoma U2OS cells. (**A**) 2D-group cell migration ability was determined using wound healing assays. Phase contrast images of the wound gaps between two monolayer cells were captured at 0, 12, 18 and 24 h. (**B**) Percentage of wound closure was quantified by the following calculations: $Area_0$-$Area_t$/$Area_0$ x 100%. Bars represent the mean ± SEM. ***P < 0.001 by one-way ANOVA.
